# The Medical Student’s Vade Mecum

**Published:** 1852-06

**Authors:** 


*The Medical Student's Vade Mecum; a compendium of Anatomy, Physiology, Chemistry, Materia Medica and Pharmacy, Surgery, Obstetrics, etc., etc.* By George Mendenhall, M.D., Lecturer on Obstetrics in the Medical Institute of Cincinnati; Member of the Am. Med. Ass., etc., etc. Third edition, revised and greatly enlarged, with two hundred and twenty-four engravings: pp. 690, 12mo.: Lindsay & Blakiston, Phil., 1852. From the publishers, through S. C. Griggs & Co.

The publication of a third edition of the work with the above long and comprehensive title, is, to say the least, evidence that it finds purchasers, and for the same reason, doubtless, that schools destitute of the requisite means for giving clinical and anatomical instruction, are about as successful in obtaining students as those having at their command well filled hospitals and an abundance of anatomical material.

The object of the book, embracing as it does all the departments of medicine in a single volume, is to communicate knowledge in the manner and spirit of the age, by telegraphic and railroad speed, and with the least possible expenditure of time and labor.

Modern progress will doubtless continue to "battle" successfully "with time and space" till their influence upon the mere physical world is less even than at present; but we doubt much if any method has been, or will be, discovered by which to make to order, and in a day, good physicians.

We regard, therefore, as worse than useless a mode of teaching such as is adopted in this work, of which the following quotations are examples:

Anatomy.—

Q. What is Anatomy? A. The science of organization.

How is it divided? Into *Vegetable* and *Animal.*

How is Animal Anatomy divided? Into *Human* and *Comparative.*

What is Human Anatomy? The Anatomy of man.

What is Comparative Anatomy? The Anatomy of all other animals except man.

How is Human Anatomy divided? Into *Descriptive*, or *Special*, *Surgical*, *General*, and *Pathological.*

What is Descriptive or Special Anatomy? That which describes the form, size, position, and connections of organs in a healthy condition of the body.

What is Surgical Anatomy? That which treats of the relation of one part to another, and has also been termed *Regional Anatomy.*

What is General Anatomy? That which treats of the structure of the simple tissues of the body.

What is Pathological Anatomy? That which relates to the diseased structure of the organs.

Of Disease.—

How may diseases be *divided?* Into *organic* and *functional.*

What is the character of the *first* class? There is a change which is appreciable by our senses, in the structure of one or more organs.

What is the character of the *second* class? The disordered function is not attended by any appreciable lesion.

Each of them may again be divided into *acute* and *chronic*, and *general* and *local affections.*

Which of the two classes is generally the more *fatal? Organic* diseases; but several of the *functional* are extremely mortal, as tetanus and hydrophobia.

What is meant by *local organic diseases?* Those in which the important symptoms are local, and are nearly in proportion to the anatomical lesions found after death, when it takes place. They may be acute or chronic.

What are the *general organic diseases?* They are often chronic, as tuberculous and cancerous diseases. The acute are certain epidemic dysenteries, fevers, scurvy, and gangrene. Tubercles are, however, sometimes acute. H.

